# A simple optical flow model explains why certain object viewpoints are special

**DOI:** 10.1098/rspb.2024.0577

**Published:** 2024-07-10

**Authors:** Emma E. M. Stewart, Roland W. Fleming, Alexander C. Schütz

**Affiliations:** ^1^ School of Biological and Behavioural Sciences, Queen Mary University London, London E14NS, UK; ^2^ Department of Experimental and Biological Psychology, Queen Mary University London, London E14NS, UK; ^3^ Centre for Brain and Behaviour, Queen Mary University London, London E14NS, UK; ^4^ Department of Experimental Psychology, Justus Liebig University Giessen, Giessen 35394, Germany; ^5^ Centre for Mind, Brain, and Behaviour (CMBB), University of Marburg and Justus Liebig University Giessen, Giessen 35032, Germany; ^6^ General and Experimental Psychology, University of Marburg, Marburg 35032, Germany

**Keywords:** viewpoint perception, perspectival appearance, three-dimensional object perception

## Abstract

A core challenge in perception is recognizing objects across the highly variable retinal input that occurs when objects are viewed from different directions (e.g. front versus side views). It has long been known that certain views are of particular importance, but it remains unclear why. We reasoned that characterizing the computations underlying visual comparisons between objects could explain the privileged status of certain qualitatively special views. We measured pose discrimination for a wide range of objects, finding large variations in performance depending on the object and the viewing angle, with front and back views yielding particularly good discrimination. Strikingly, a simple and biologically plausible computational model based on measuring the projected three-dimensional optical flow between views of objects accurately predicted both successes and failures of discrimination performance. This provides a computational account of why certain views have a privileged status.

## 1. Introduction

When asked to imagine a familiar object, most people find themselves picturing the object from particular viewpoints that are especially informative or qualitatively distinct from other views [1]. Yet, despite decades of research on visual object perception, fundamental questions remain about why certain views of objects are special. There is a confusing array of terms and ideas related to different kinds of views, including ‘canonical’ [2–6], ‘accidental’ or ‘non-accidental’ [7–9], ‘generic’ [10–12] or ‘cardinal’ (or being aligned along a cardinal axis [13–17]). Here, we sought a computational framework for understanding why some views are privileged in object perception. We show that a simple computational model, based on optical flow [18], can accurately predict the costs and benefits of viewing both familiar and novel objects from certain perspectives. The model provides a straightforward quantitative account of how the visual system determines which views of objects are particularly important, based on regularities in object geometry and the two-dimensional visual information that is projected onto the retina.

Viewpoints play an important role in object perception and can influence how we perceive, remember and recognize an object. Here, we will consider three specific geometrically and qualitatively distinct viewpoints: (i) canonical viewpoints [19], which tend to be oblique views where most of the surface of the object is visible [2,20]; (ii) end-on cardinal viewpoints, when an object is viewed so that the viewpoint with the smallest width-to-length ratio is aligned along the viewing axis (e.g. viewing a pig front on); and (iii) conversely the flat sides of objects, where the largest width-to-length ratio is aligned along the viewing axis (like the side of a pig) [21,22]. Viewing an object from one of these special viewpoints can benefit object recognition and recall [1,3,5,6] and can result in longer inspection times of the object [23]. For example, the canonical viewpoints of an object might convey the most information about its overall identity and are the best views for object recognition [19], and conversely, the flat viewpoints are the most perceptually stable or provide the least amount of visual change if the object were to rotate a little [21,22]. People are also better at performing viewpoint discrimination tasks from some viewpoints. Previous work has attempted to quantitatively define the transition between qualitatively distinct views [22] as ‘visual events’ and found that when an object is rotated across one of these visual events, discrimination performance is higher. Such discrimination benefits were found to be particularly strong for the front and back of familiar objects, particularly when the objects were symmetrical and/or had orientation-specific features such as linear contours [21].

We reasoned that we could use these geometric regularities in object viewpoint discrimination to provide a quantitative prediction of qualitatively distinct viewpoints, derived from the extent to which points on the object shift in the image when viewpoint (or equivalently, object pose) changes. To do this, we used a simple model inspired by optical flow computations, which was recently shown to capture the non-uniformities and geometric regularities in object viewpoint perception [18]. Specifically, the model assumes that given a pair of views of an object, the visual system (i) identifies corresponding points on the objects’ surface across the two poses, (ii) estimates the vectors in three-dimensions (3D) between these corresponding points, (iii) projects the vectors into the image plane from the current perspective, and (iv) takes the average length of these vectors as a measure of the difference between the two poses. We find that this approach provides a quantitative account of the differences between views and thus a quantitative framework for understanding what makes certain object viewpoints qualitatively special.

## 2. Results

### (a) Experiment 1: viewpoint discrimination judgements

**Figure 1.**
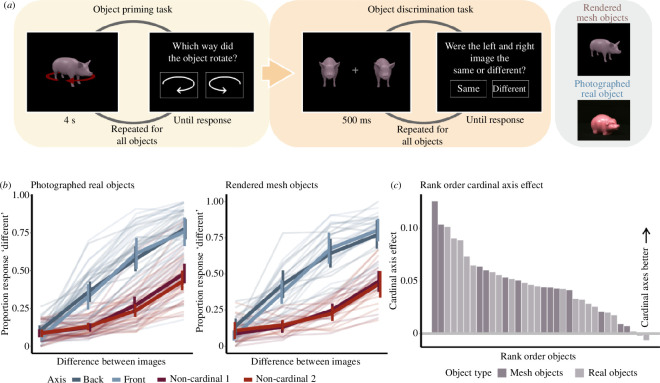
(*a*) Participants first completed a priming task: each object rotated for for 4 s; participants indicated rotation direction via button press. Participants then completed a pose discrimination task. Two viewpoints of an object were shown: the cardinal/non-cardinal viewpoint and a viewpoint offset by 0°, ±5°, ±10° or ±15° rotation. Participants had to indicate whether the two viewpoints were the same or different. Photographed real objects and rendered mesh objects were tested in two separate experiments. (*b*) Human discrimination performance for photographed (left) and rendered (right) objects. Pale lines: individual participants; bold lines: means; error bars indicate standard errors. (*c*) The cardinal axis effect for each object, rank-ordered. This was calculated as the slope between performance at 0° and 5° difference. Real objects are depicted in light purple and mesh objects in dark purple.

We reasoned that if the proposed optical flow model can be used to unify previous qualitative findings on cardinal viewpoints, it should first be able to predict discrimination benefits at cardinal (front and back) versus non-cardinal (oblique) viewpoints [21,22]. In two online experiments, we collected human discrimination judgements for cardinal and non-cardinal viewpoints for 21 photographs of real objects from the Amsterdam Library of Object Images (ALOI) [24] and 13 rendered mesh objects (see §4). In an initial object priming block, participants were shown a video of each of the objects rotating for 4 s and were asked to report the direction of rotation. This task aimed to prime participants to think about the 3D rotational nature of the objects in the subsequent task. Results from this block were only used for participant exclusion (one participant for this criterion) and were not analysed further. In the subsequent object discrimination block, two viewpoints of the same object were displayed on either side of a central cross for 500 ms: the base viewpoint was either a cardinal or a non-cardinal viewpoint, and the rotated viewpoint was offset from the base view by seven possible rotation levels (0°, ±5°, ±10°, ±15° rotation around the vertical axis). Participants indicated whether the two viewpoints were the same or different by clicking an on-screen button (figure 1*a*).

To analyse the responses, for each object and rotation level, we calculated the proportion of responses where participants indicated that the two views were the same (figure 1*b*). Results showed a striking difference in performance between cardinal and non-cardinal axes for both the photographs of real objects and the rendered mesh objects. A generalized least squares (GLS) regression model (performance ~ rotation_level (0, 5, 10, 15) × axis_type (cardinal, non-cardinal) × image_set (real, rendered)) demonstrated a significant effect of rotation level: *F*_1,536_ = 734.76, *p* < 0.0001; axis type: *F*_1,536_ = 127.88, *p* < 0.0001; and the interaction between rotation level and axis type: *F*_1,536_ = 28.92, *p* < 0.0001. There was no significant main effect of image set: *F*_1,536_ = 0.24, *p* = 0.63; and no other interactions were significant. This demonstrates that humans are better at discriminating objects rotated around cardinal versus non-cardinal axes for both real and rendered objects. These cardinal axes would, therefore, seem to be analogous to the ‘visual events’ postulated by Tarr & Kriegman [22].

**Figure 2.**
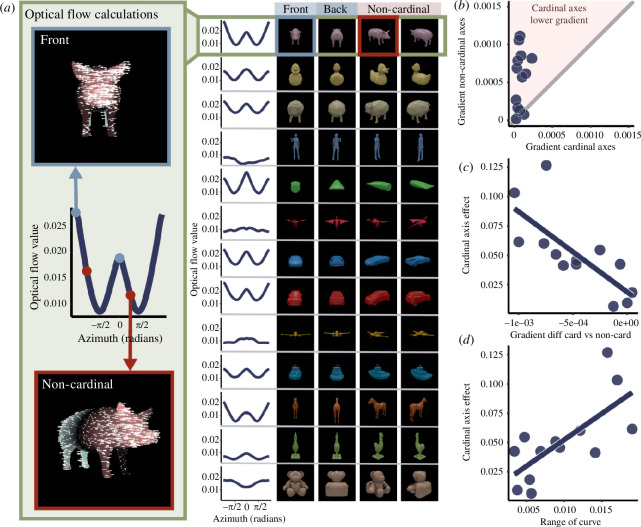
(*a*) Optical flow model. For each view of each mesh object, the optical flow was calculated between that view and the next view, resulting in an optical flow curve (left, middle). Left top and bottom show example optical flow outputs for the front and non-cardinal axes of one object (pink indicates rightwards motion and green indicates leftwards motion). Right panel shows optical flow curves for each object, with front, back and non-cardinal viewpoints of each object. (*b*) Comparison of the gradient of cardinal versus non-cardinal axes on the optical flow curve. Dots represent individual objects. The pink shaded area represents objects where the optical flow gradient was lower for cardinal than non-cardinal axes. (*c*) The cardinal axis effect (difference in slope between 0° and 5° offset, as in figure 1*c*) was predicted by the gradient difference between cardinal and non-cardinal axes on the optical flow curve. (*d*) The cardinal axis was predicted by the range of the optical flow curve.

The results also showed that there was some variability in performance between objects. To quantify the discrimination benefit for cardinal versus non-cardinal axes for a particular object, we calculated the ‘cardinal axis effect’ as the difference in the slope between ‘response different’ judgements for 0° and 5° rotation levels for cardinal versus non-cardinal axes, as this was the difference at which the most variability was observed between objects. The higher the slope for an object/axis, the more discriminable it is. Figure 2*c* shows that the cardinality effect was stronger for some objects than others, for both real and mesh objects, with 32/34 objects showing a perceptual discrimination benefit for cardinal versus non-cardinal viewpoints. This variability in the cardinal axis effect allowed us to use a model to investigate to what extent we could predict the cardinal axis effect for each object, and whether features of our model predictions might predict the magnitude of this effect.

### (b) Optical flow model predicts cardinal viewpoints

We used an optical flow model to compute viewpoint dissimilarity for every viewpoint around each object. In brief, the model measures how much points on objects shift in the image as the viewpoint changes. Specifically, given a pair of poses of an object, we compute the 3D vectors between corresponding surface points and then estimate the mean length of these vectors when projected into the two-dimensional (2D) image plane. This model has been shown to capture viewpoint-related variations in mental rotation (18), and we reasoned that it may be able to predict both cardinal and non-cardinal axes within objects, as well as the relative degree of the cardinal axis effect across objects. For each of the 72 rendered viewpoints, we calculated the ground-truth optical flow vectors produced as the object rotated towards the next viewpoint. The model prediction for this viewpoint was taken as the mean of the absolute length of these vectors (see Stewart *et al.* [18] for further details). We thus obtained an ‘optical flow curve’ for each object (figure 2). We calculated the gradient of this flow curve at the tested viewpoints and the slope of the curve between the tested base viewpoint (front, back and non-cardinal) and each offset viewpoint. Different objects had different optical flow curve profiles and in particular varied in the range of the curve. We therefore also calculated the range (max–min) of the curve for each object.

The gradient of the optical flow curve predicted cardinal versus non-cardinal axes, with cardinal axes having a lower gradient than non-cardinal axes (*t*(12) = −4.58, *p* = 0.0006, Cohen’s *D* = 1.27 (large effect size); figure 2*b*). The optical flow model could also predict human discrimination performance in two ways. We first looked at whether the cardinal axis effect could be predicted by the gradient of the optical flow curve (figure 2*c*). A simple linear regression model revealed that the model gradient accurately predicted the human discrimination performance (*F*_1,50_ = 14.47, *p* = 0.00039). Second, we tested whether the cardinal axis effect could be predicted by the difference in gradient (figure 2*d*) and by the magnitude of optical flow change across the entire optical flow curve (range of the curve). Simple linear regression models showed a significant effect of gradient difference (*F*_1,11_ = 14.02, *p* = 0.0033) and of curve range (*F*_1,11_ = 11.21, *p* = 0.0065). These results indicate that the gradient of the optical flow curve is predictive of whether a viewpoint is cardinal or not, providing for the first time a straightforward quantitative predictor for these qualitatively special views of familiar objects.

### (c) Experiment 2: the front of novel objects

**Figure 3.**
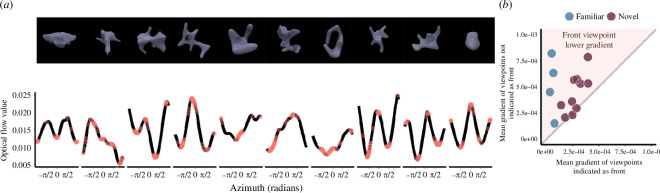
(*a*) Tested novel objects (top) with the corresponding optical flow curves (bottom). Novel objects are shown from the viewpoint with the highest number of ‘front’ responses. Individual points on the curve represent viewpoints that were marked as the ‘front’ of the object. (*b*) Scatter plot representing the mean gradient for each object across participants at viewpoints selected as ‘front’ compared with viewpoints that were not selected as ‘front’. Novel objects are represented in purple, and the four familiar objects tested are shown in blue for comparison. The shaded pink area represents where the viewpoints selected as ‘front’ have a lower gradient than non-front viewpoints.

The objects tested in experiment 1 were all easily recognizable objects, and factors such as familiarity and the geometric properties of the shapes themselves (e.g. symmetry and elongation) might have influenced performance. Thus, while model predictions correlated with the cardinal axis effect for symmetrical, elongated, real objects, the findings may not generalize to novel objects with less regular elongation and symmetry [9]. We therefore created 10 non-meaningful mesh objects with both regular and irregular optical flow curves, and in an online experiment we verified that these objects were on average rated as non-familiar (see §4b(i) for details of object-familiarity ratings). Objects were created to have varying levels of symmetry and elongation, and to have a more heterogenous pattern of optical flow predictions than the familiar objects in experiment 1. We then conducted a separate online experiment and asked a new sample of 50 online participants to rotate each of the 10 novel objects, plus four of the familiar mesh objects from the previous experiment (pig, duck, small car and figure), so that the front of the object was facing towards the participant. For each object, we examined where the responded ‘front’ angles lay on the optical flow prediction curve. In general, responses tended to cluster around the peaks and troughs of the optical flow curve (figure 3), and on average the viewpoints that were indicated as being a ‘front’ had lower gradients than the viewpoints that were never indicated as being the front (Wilcoxon test *z* = 2, *p* = 0.00037, *r* = 0.54; strong effect size). This demonstrates that, while there is naturally more variability in where the actual front of the object is considered to be, even for novel, non-symmetrical objects, the optical flow model was predictive of which viewpoints may be considered to be candidate ‘front’ views of these objects.

## 3. General discussion

**Figure 4.**
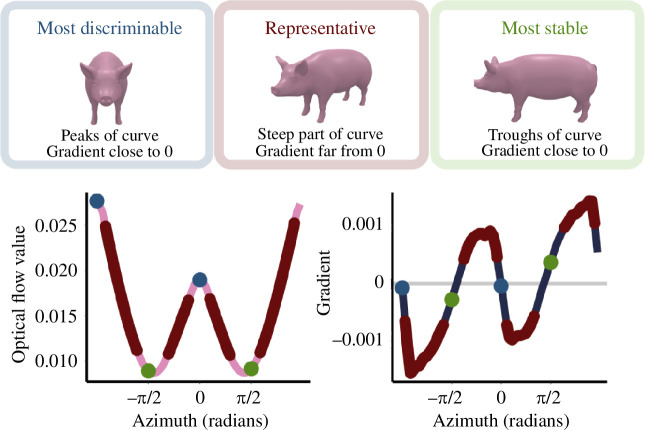
This model can quantitatively predict and constrain which viewpoints of a 3D object are qualitatively meaningful using the predicted dissimilarity curve and the gradient of this curve.

Our results suggest that the optical flow model can explain variability in viewpoint discrimination and identify qualitatively distinct viewpoints. Participants were better at discriminating between two viewpoints separated by 5° rotation when one of those viewpoints was a so-called cardinal axis, compared with when it was an oblique view of the object. The magnitude and variability in this perceptual discrimination advantage could be predicted by the gradient of an optical flow model that computes the magnitude of the 2D displacement vectors that would be produced if the object were to rotate from one viewpoint to the next. Remarkably, this model could also predict viewpoints that were more likely to be labelled as ‘front’ for novel, unfamiliar objects. This model can, therefore, capture quantitative geometrical relationships between viewpoints and predict which viewpoints stand out as particularly significant for the observer. Our findings suggest that the method works for familiar, unfamiliar, regular and irregular objects. As figure 4 shows, viewpoints that may be considered the most discriminable (front and back); most stable (sides); and ‘typical’, ‘generic’ or ‘representative’ (oblique; see electronic supplementary material) can be constrained using the model output curve (optical flow value/predicted perceived dissimilarity) and the gradient of this curve. Thus, a simple, quantitative account based on the projected spatial shifts of visible surface points provides a computational framework for object viewpoint perception.

It is likely that geometrical regularities across natural objects contribute to a form of statistical learning about object geometries. For example, in most quadrupedal animals, the front and back of the animal are narrower and more symmetrical than the side-on view. While such statistical learning about object categories and identity may account for familiarity effects in object recognition [2], learning about the geometry of objects may also aid in identifying important viewpoints for unfamiliar objects. The results of experiment 2 suggest that participants may extrapolate learned geometrical regularities of cardinal viewpoints experienced in the real world and use these statistical regularities to determine the cardinal viewpoints of novel objects. As with many other object features [25–29], the visual system seems to use knowledge of objects in the world to form priors about [30] or estimate latent variables underlying [31] the proximal information about the geometry of distinctive object viewpoints.

These results also allow us to reflect on the nature of object viewpoint representations in relation to their true distal form as opposed to our 2D proximal experience of them. We recently suggested, based on the model also used in the current study, that the computations underlying the 3D mental rotation of objects rely on a ‘mental rendering’ of the imagined object, as if predicting its 2D proximal appearance [18]. Here, we suggest that the comparison and categorization of 3D object viewpoints might then rely on similar 3D to 2D computations. Interestingly, in this experiment, even though participants were primed on the 3D nature of the stimulus by being shown the object rotating in space, the model based on distances in 2D is still predictive of human performance and reflects previous findings that a 2D representation may underpin a 3D viewpoint discrimination [18,32,33]. In experiment 2, the selection of the ‘front’ viewpoint compared with all other viewpoints arguably requires a 3D representation of the object as a whole, yet even in this case responses corresponded to geometric regularities predicted by the 2D model. As a result, the model also provides a route into understanding the origin of putative effects of the ‘perspectival’ appearance of objects [34–36]—such as the perceived ‘elliptical nature’ of a coin seen slanted in depth—in the context of theories of vision that assume perceptual constancies [37–40]. Specifically, we suggest that even when a 3D object structure is estimated perfectly, comparisons between objects are made in terms of the estimated or predicted changes in the proximal stimulus involved in transforming one view to another. We speculate that representations that express changes in terms of proximal stimulus quantities are particularly useful in learning to see a 3D object in the absence of ground-truth data about its physical state. Specifically, we suggest that learning to accurately predict changes in the proximal stimulus teaches the visual system deep knowledge about the distal forms that produce such changes [31,41]. Thus, paradoxically, retaining a representation of ‘perspectival aspects’ of objects may be a key step in learning to see them as 3D in the first place. Our findings suggest that humans use proximal information about an object’s geometry to make judgements about an object’s pose, and more importantly, they use this information to learn regularities about qualitatively special viewpoints.

## 4. Material and methods

### (a) Participants

In total, 300 participants completed the study. These were distributed between the experiments as follows. Experiment 1, real objects: 50 participants (24 female; age range 19–59, mean age 29.9 years (s.d. 1.3)); experiment 1, rendered mesh objects (*n* = 50, female = 27, undisclosed sex = 1, mean age = 31.4 years (s.d. 10.4)). Experiment 2, familiarity ratings 1 (*n* = 50, 18 female, mean age = 27.8 years (s.d. 5.9)); experiment 2, familiarity ratings 2 (*n* = 50, 16 female, mean age = 27.9 years (SD 8.5)). Experiment 2, front judgements: 50 participants (*n* = 50, 19 female, mean age = 30 years (s.d. 8.7)). Experiment 2, representative judgements: 50 participants (*n* = 50, 28 female, mean age = 41.9 years (s.d. 12.7)). Participants participated online and were recruited through Prolific (https://www.prolific.com/). Experiments were approved by the University of Marburg local ethics committee (approval number 2015-35 k) and the University of Giessen local ethics committee (approval number 2020-0033) and were conducted in accordance with the Declaration of Helsinki (1964).

### (b) Stimuli

#### (i) Experiment 1—photographed real objects

Photographs of real objects were taken from the ALOI [24]. This library contains 1000 photographs of real-world objects, photographed from 72 viewpoints separated by 5° of horizontal rotation. In a previous study, we collected human judgements about which viewpoints of these objects corresponded to a number of axis labels (front, back, left, right, prototypical; see [42] for details on data collection). These data gave us a distribution of angular responses for each axis label for each object. For this study, we chose 21 objects that had clearly defined cardinal viewpoints: for each object and viewpoint label, we calculated the mean resultant length of participant responses and used this to select objects for which there was high agreement between participants for all cardinal viewpoints. The cardinal viewpoint for a specific label was then taken as the circular mode of the responses for that label. Non-cardinal viewpoints were taken as those that were midway between two cardinal viewpoints (i.e. front and left) but were not reported to be the prototypical or any other viewpoint.

#### (ii) Experiment 1—rendered mesh objects

We repeated the experiment with mesh objects so that we could apply the mesh-based optical flow model. We selected 13 mesh objects that were as similar as possible to the original ALOI objects in both shape and semantic meaning. Meshes were either freely available online or selected from Evermotion (https://evermotion.org). These meshes were rendered in Blender [43] from 72 viewing angles separated by 5° horizontal rotation, using lighting and camera distance that were analogous to the ALOI objects [24]. For these objects, the front was defined by the researchers (and was analogous to the front of the photographed objects) and the same angular rotations relative to the front as in the photographed objects were used to define non-cardinal viewpoints. Objects were rendered in pretty colours chosen by the researchers.

#### (iii) Experiment 2—unfamiliar objects

We created 30 semantically non-meaningful mesh objects using Mathematica (https://www.wolfram.com/mathematica/) and Blender [43] and chose the 10 most unfamiliar objects (see §6b(ii) for details). These objects were rendered from the same viewing angles, with the same lighting conditions as in experiment 1. The four familiar objects from experiment 1 were re-rendered in the same colour as the unfamiliar objects.

## 5. Procedure

### (a) Experiment 1

Experiment 1 (photographed real objects) was pre-registered at https://aspredicted.org/ (#67124), and the experiment using the rendered mesh objects followed the same protocol. The experiments were conducted online and programmed with custom-written software using jsPsych [44]. Each participant completed both an object priming task and a perceptual discrimination task. The object priming task aimed to familiarize participants with the 3D nature of the objects and to prime them to think about the objects rotating through viewpoints. Participants viewed a video of each object rotating through 360° for a duration of 4 s, either clockwise or counterclockwise. Participants had to indicate the direction of rotation via button press. Participant responses to the priming task were only used for excluding participants and were not analysed further. In the perceptual discrimination task, participants were presented with two views of an object on either side of a central fixation cross, for 500 ms. They then indicated via button press whether the views were the same or different. These views were defined by four conditions, tested in two separate experiments—experiments 1 and 2 (part 1): front ± 0°, 5°, 10°, 15°; non-cardinal angle 1 ± 0°, 5°, 10°, 15°. Experiments 1 and 2 (part 2): back ± 0°, 5°, 10°, 15°; non-cardinal angle 2 ± 0°, 5°, 10°, 15°. Every participant saw all objects at both cardinal and non-cardinal viewpoints, with randomized viewpoint offset difference levels.

### (b) Experiment 2

#### (i) Familiarity ratings

To choose the ratings, participants saw a video of each object rotating and indicated how familiar they found the object to be, using a 6-point slider ranging from ‘very unfamiliar’ to ‘very familiar’. The video looped to give the appearance of a continuously rotating object until a response was given. Four of the objects from experiment 2 were included as catch and comparison trials (pig, car, figure, duck). Mean familiarity ratings were calculated, and objects were rank ordered by familiarity. We chose 10 objects for the cardinal axis rating task, accounting for low familiarity ratings, varying levels of symmetry and varying optical flow curve profiles. To confirm that these chosen novel objects were perceived as being unfamiliar, we ran a second online experiment with the same procedure, where participants only saw these 10 unfamiliar objects and the 4 familiar objects (see electronic supplementary material).

#### (ii) Cardinal axis ratings

In an online experiment, participants were instructed to rotate each object using the left and right arrows on the keyboard until the object was facing toward the ‘front’.

### (c) Exclusions

In experiment 1, trials were excluded if the reaction time was less than 300 ms or more than 5000 ms or if performance was less than 75% correct on either the training or the main task. In experiment 2, for the familiarity ratings, trials were excluded if the reaction time was less than 300 ms or more than 5000 ms, or if they rated any of the real objects (pig, figure, car) as having a familiarity rating of less than three out of six. For the front ratings, trials were excluded based on the same reaction time criteria, and additionally if the ‘front’ response of familiar objects was not within 45° of the veridical front. Overall, 1.2% of trials were excluded for experiment 1 (photographed real objects) and 1.19% for the rendered mesh objects. For experiment 2 (familiarity ratings), no trials were excluded; for experiment 2 (front ratings), three participants were excluded, and no trials were excluded from the remaining participants.

## 6. Analyses

### (a) Model

We used a ground-truth optical flow computation [18], which has previously been found to predict human performance for viewpoint dissimilarity judgements for block-sequence 3D rendered objects. The model predicted the amount of 2D optical flow information that would be produced as the object rotated by 5° from one viewpoint to the next, by calculating the mean of the absolute horizontal and vertical displacements of every visible vertex in the underlying object mesh. Displacements for vertices visible from one viewpoint but not the rotated viewpoint (unmatched points) are not included in model calculations.

### (b) Cardinal axis effect

To calculate the cardinal axis effect, we only took the slope of performance difference between 0° and 5° degree displacements for the following reasons: (i) this is the most difficult discrimination level we tested; (ii) larger discrimination levels (especially 15°) become trivial; and (iii) there is not necessarily a linear increase in performance between 0°, 5°, 10° and 15° displacements. For some objects, there is a very small performance difference between 0° and 5° displacements; for others, there is a larger difference in performance. Such between-object differences in sensitivity to small displacements are lost if larger displacements are included in the cardinal axis effect calculations.

### (c) Statistical analyses

All statistical analyses were conducted in R [45]. Linear regression models were conducted using base R. GLS models were conducted using the package nlme [46] and were implemented where a linear model would otherwise have a heterogonous variance across different levels of a factor. Model comparisons were used to determine the best-fitting variance structure (different variance allowed for different factor levels).

## Data Availability

Data and stimuli are available at [47]. Supplementary material is available online [48].
